# Pioglitazone Modulates p65-Mediated Mitochondrial Bioenergetics: Implications for Acetaldehyde-Induced HIV Replication in Alveolar Macrophages

**DOI:** 10.3390/biom15121737

**Published:** 2025-12-13

**Authors:** Moses New-Aaron, Sarah Chang, Xian Fan, Ashish Mehta, Sara C. Auld, Bashar S. Staitieh, Michael Koval, Samantha M. Yeligar

**Affiliations:** 1 Division of Pulmonary, Allergy, Critical Care and Sleep Medicine, Department of Medicine, Emory University School of Medicine, Atlanta, GA 30322, USA; moses.new-aaron@emory.edu (M.N.-A.); sarah.s.chang@emory.edu (S.C.); xfan@emory.edu (X.F.); amehta4@emory.edu (A.M.);; 2 Atlanta Veterans Affairs Health Care System, Decatur, GA 30033, USA; 3 Grady Memorial Hospital, Atlanta, GA 30303, USA

**Keywords:** mitochondrial hyperactivation, EcoHIV, acetaldehyde generating system, inflammation, alveolar macrophages, alcohol, pioglitazone, nuclear factor kappa B p65

## Abstract

Alcohol misuse is twice as prevalent among people living with HIV (PWH), and this increases the risk of pulmonary complications even in those receiving antiretroviral therapy. Our prior work showed that the alcohol metabolite, acetaldehyde, activates nuclear factor kappa B p65 (p65), leading to HIV replication and interleukin (IL)-1β activation in alveolar macrophages (AMs). Since the aforementioned processes are energy-demanding, which conversely impair mitochondrial functions, we hypothesized that acetaldehyde-induced p65 drives AMs to a mitochondrial hyperactive state to promote HIV replication and IL-1β release and induces oxidative stress and mitochondrial dysfunction. Since we found pioglitazone (PIO) to be a negative regulator of p65, we postulate that PIO suppresses HIV replication and IL-1β activation in AMs by restricting p65-induced mitochondrial hyperactivation. Murine AMs were exposed to acetaldehyde via the acetaldehyde generating system (AGS) and infected in vitro with EcoHIV, a chimeric ecotropic HIV construct. AGS + EcoHIV activated p65, resulting in enhanced ATP-linked mitochondrial respiration, proton leak, non-mitochondrial respiration and the generation of reactive oxygen species (ROS) in AMs. Inhibition of mitochondrial ATP synthesis with low-dose oligomycin attenuated AGS-induced HIV replication and AGS + EcoHIV-induced IL-1β release from AMs. PIO treatment, which attenuated AGS-induced p65 activation, suppressed proton leak, non-mitochondrial oxygen consumption, ROS, and IL-1β and p24 release. While p65-induced mitochondrial hyperactivation represents AMs’ adaptive response to the energy demands imposed by HIV replication and proinflammatory activation when exposed to acetaldehyde, PIO treatment may offer a novel therapeutic strategy to restore adequate mitochondrial bioenergetics in the AMs of PWH who misuse alcohol.

## 1. Introduction

Despite the lack of cure for human immunodeficiency virus (HIV), tremendous success has been achieved in managing disease progression in people living with HIV (PWH) through antiretroviral therapy (ART) [1]. Modern ART makes achieving suppressed viremia and increased longevity more attainable [2]. However, increased longevity among PWH increases the risk of age-related chronic diseases, including infectious and non-infectious respiratory complications [3]. Respiratory illnesses are a significant concern in HIV, as they are the leading cause of hospitalization and non-AIDS-related mortality [4,5,6,7,8]. A recent study among PWH demonstrated that using ART for at least 4 years was associated with suppressed mortality from all causes except substance use and respiratory illnesses [8]. This suggests that HIV-related respiratory complications in the context of substance use, such as alcohol misuse, are a critical issue in the era of ART.

The lung consists of several HIV-permissive cells, including alveolar macrophages (AMs). AMs, which make up more than 80% of the bronchoalveolar lavage fluid (BALF), are at risk of HIV infection [9]. While HIV is cytopathic to other immune cells, HIV persists in AMs, which live longer than other lung immune cells, thereby supporting AMs as an HIV reservoir even in individuals using ART [10]. Agents associated with ART are detected in the BALF of PWH on ART [11]. However, it remains unclear whether ART’s biodistribution to the alveoli maintains the same potency as it has in the plasma, since several studies also detected HIV in the BALF of PWH on ART [12,13,14]. The persistence of HIV in AMs could contribute to HIV driving chronic inflammatory responses in the lungs, hence increasing the risk for HIV-associated respiratory complications [15]. This increased risk is exacerbated by other comorbidities like alcohol misuse, which is frequent among PWH [16]. We recently observed elevated respiratory symptom burden and pulmonary dysfunction among PWH with a history of excessive alcohol consumption [17]. Recently, we also demonstrated that AMs in individuals with alcohol use disorder (AUD) are exposed to the toxic alcohol metabolite, acetaldehyde, which likely contributes to the detrimental effects of alcohol in the lungs of PWH, such as increased HIV replication and chronic inflammatory responses [18].

HIV replication and the activation of IL-1β, a proinflammatory cytokine, are energy-consuming processes [19,20]. HIV usurps the cellular bioenergetic pathway of many immune cells for its proliferation and downstream effects [21] but whether this occurs in AMs was unknown. The nuclear factor kappa B p65 (p65), which is activated by acetaldehyde [18], reprograms the energy metabolism pathway of myeloid cells during viral infections [22]. However, the links between the p65 signaling pathway, HIV replication, IL-1β activation, and mitochondrial bioenergetics in HIV-infected AMs within the context of alcohol metabolism are unknown. Understanding the relationship between these processes is crucial for delineating molecular pathways that may underlie acetaldehyde-induced HIV replication and IL-1β activation in AMs.

Previous studies have shown that alcohol metabolism significantly alters mitochondrial phenotype, leading to a state of mitochondrial hyperactivity characterized by increased mitochondrial respiration, proton leak, and the generation of reactive oxygen species (ROS), ultimately resulting in mitochondrial dysfunction [23,24]. Since AMs challenged by alcohol metabolism and HIV independently induce oxidative stress [18,25,26], we expected that mitochondrial hyperactivity would characterize the mitochondrial energy profile of AMs exposed to these stressors. Therefore, we hypothesize that acetaldehyde-induced p65 reprograms AM mitochondria to increase bioenergetics for HIV replication and IL-1β release, while also impairing mitochondrial function.

Given the physiological relevance of mitochondrial bioenergetics, mitochondrial dysregulation in AMs undoubtedly plays a significant role in contributing to pulmonary dysfunction [25,27,28]. Although older ART disrupts the mitochondria [29,30,31], modern ART is far less toxic to mitochondria. However, some studies have reported organ-specific residual effects of modern ART on the mitochondria [32,33,34] beyond the effects of HIV infection and alcohol misuse. Therefore, this study will exclude treatment of AMs with ART to specifically delineate the impact of HIV and alcohol metabolism on AMs. Furthermore, therapeutic targets that will inhibit HIV replication and proinflammatory activation, along with the enhancement of mitochondrial functions in AMs, may improve pulmonary complications, among vulnerable populations, such as individuals with AUD. Peroxisome proliferator-activated receptor gamma (PPARγ), a negative regulator of p65 activation, is known to attenuate HIV replication and proinflammatory changes while restoring mitochondrial bioenergetics in AMs [18,25,35]; thus, an agonist of PPARγ, such as pioglitazone (PIO), may be beneficial. In this study, we investigated the effects of acetaldehyde-induced p65 on mitochondrial hyperactivation and examined whether PIO suppresses acetaldehyde-induced p65 activity and improves mitochondrial bioenergetics, thereby attenuating HIV replication, IL-1β activation, and ROS activation in AMs.

## 2. Materials and Methods

### 2.1. Reagents and Antibodies

We purchased RPMI-1640 medium from Corning (Corning, NY, USA) and fetal bovine serum (FBS) from ScienCell Research Laboratories (Carlsbad, CA, USA); gentamicin was purchased from ThermoFisher Scientific (Waltham, MA, USA); sodium bicarbonate and 2-mercaptoethanol were purchased from Sigma-Aldrich (St. Louis, MO, USA); PIO was purchased from USBiological Life Sciences (Salem, MA, USA); JSH-23, Cat # HY-13982 was purchased from MedChem Express (Monmouth Junction, NJ, USA); MitoSOX™ Mitochondrial Superoxide Indicator and Amplex™ Red Hydrogen Peroxide/Peroxidase Assay Kit were purchased from Thermo Fisher Scientific (Waltham, MA, USA). TRIzol was purchased from Life Technologies (Carlsbad, CA, USA); iTaq Universal SYBR Green One-Step kit from Bio-Rad (Hercules, CA, USA); and NuPAGE 10% Bis-Tris gel was purchased from Invitrogen (Carlsbad, CA, USA). Primary antibodies used were (a) mouse monoclonal: anti-p24: sc-69728 (Santa Cruz Biotechnology, Dallas, TX, USA) and (b) Rabbit polyclonal anti-p65: 8242; anti-cleaved IL-1β: 63124 (Cell Signaling Technology, Boston, MA, USA), anti- nicotinamide adenine dinucleotide phosphate oxidase (NOX)4: 14347-1-AP (Proteintech, Rosemont, IL, USA). Secondary antibodies were IRDye 680RD goat anti-rabbit, C50317-02; IRDye 680RD donkey anti-mouse, C50520-02 (LICORbio, Lincoln, NE, USA); Goat anti-Mouse IgG (H + L) Cross-Adsorbed Secondary Antibody, Alexa Fluor™ 488 A-11001 and Goat anti-Rabbit IgG (H + L) Cross-Adsorbed Secondary Antibody, Alexa Fluor™ 555 (ThermoFisher Scientific, Waltham, MA, USA).

### 2.2. Human Samples

#### 2.2.1. Human Bronchoalveolar Lavage Fluid

As described [36], the BALF of PWH (*n* = 16) and people living without HIV, PWoH, (*n* = 10) at the primary care clinics of Grady Memorial Hospital in Atlanta, Georgia, was collected by flexible bronchoscopy of a single lobe with sequential instillation of 30 mL of normal saline. Protease inhibitor was added to the BALF before storage at −80 °C to preserve its protein content. At the time of the study, PWH had already been on ART for at least 18 months, with a cluster differentiation (CD)4 cell count exceeding 500 cells/μL and undetectable viremia. None of the participants had uncontrolled asthma, a history of cirrhosis, cardiomyopathy, chronic kidney disease, or had received immunosuppressive medications within 30 days of the study. Additionally, participants who were pregnant or breastfeeding were excluded. The Emory University Institutional Review Board (Emory IRB #00088993) approved the collection of the BALF from the participants. p24 and IL-1β were measured in BALF by ELISA.

#### 2.2.2. Primary Human AMs

As described [18], the biorepository specimens of deidentified fixed AMs were isolated from the BALF of participants recruited at the Atlanta Veterans Affairs Medical Center in Decatur, Georgia. The participants were those with AUD (*n* = 8), and those without AUD (*n* = 6), aged 26–60 years old. The use of these de-identified fixed AMs was approved by the Emory University Institutional Review Board (IRB) under IRB #00097480. Current tobacco, marijuana, and cocaine users were excluded from the study. The fixed AMs were processed and immunostained for p65 as described below in Section 2.9.

### 2.3. Acetaldehyde Generating System

Despite limited evidence about the oxidative metabolism of alcohol in AMs, several studies have substantiated AM’s exposure to acetaldehyde following alcohol exposure [37,38,39]. We recently reported the presence of malondialdehyde-acetaldehyde (MAA) protein adduct in AMs of individuals with AUD, indicating alveolar acetaldehyde exposure [18]. This corroborated previous findings of elevated acetaldehyde levels in the exhaled breath of people who misuse alcohol [40,41]. Therefore, we set up an enzymatic system that mimics the exposure of AMs to alcohol-derived acetaldehyde [42] in our in vitro studies. The system consists of 0.08% ethanol (18 mM, the legal blood alcohol concentration limit in the US) as the substrate, 2 mM nicotinamide adenine dinucleotide (NAD^+^) as the co-factor, and 0.02U yeast alcohol dehydrogenase (ADH) as the enzyme. This system continuously generates alcohol-derived acetaldehyde, and it is referred to as the acetaldehyde generating system (AGS) [18,42]. All non-AGS experimental groups were treated with media containing the same concentrations of NAD^+^ and ADH to control for their effects on the cells.

### 2.4. EcoHIV Production

HIV is a human-specific virus; therefore, murine infectious HIV studies are limited by the availability of appropriate models. The development of a chimeric ecotropic HIV model referred to as EcoHIV helps provide insight into HIV pathogenesis in rodents [43]. EcoHIV was engineered by replacing the HIV gene encoding the surface protein gp120, which is recognized by human cells, with a gp80 from a mouse leukemia virus. EcoHIV replicates in mouse macrophages and lymphocytes, inducing inflammatory responses like those observed in human cells. Infection of mice with EcoHIV has been described to replicate vital constructs of human HIV-1 infection [43]. Notably, constitutive expression of EcoHIV in mouse macrophages has been reported to persist for 16 months [44]. Here, we used EcoHIV to demonstrate HIV pathogenesis in experiments that utilized murine cells. The plasmid DNA of the EcoHIV-NDK used for this study was generously provided by Dr. Mary Jane Potash’s Laboratory at Icahn School of Medicine, Mount Sinai. We transformed and amplified the plasmid using Subcloning Efficiency DH5α Competent Cells (Cat. 18265017, Thermo Fisher Scientific, Waltham, MA, USA). Furthermore, Lenti-X^TM^ 293T cells (Cat. 632180, Takara, San Jose, CA, USA) were then transfected with the purified EcoHIV plasmid to produce the EcoHIV stocks used in our experiments.

### 2.5. In Vitro Studies

#### 2.5.1. Primary Mouse AMs

Primary mouse AMs (mAMs) were isolated from BALF drawn from 8–12-week-old male C57BL/6J mice (Jackson Laboratory, Bar Harbor, ME, USA) after euthanization. The mAMs were isolated by the centrifugation of BALF at 1800 rpm for 10 min as described [25]. The isolated mAMs were seeded in 16-well Nunc™ Lab-Tek™ Chamber culture slides (ThermoFisher Scientific, Waltham, MA, USA) and maintained in RPMI-1640 medium containing 5% FBS, 1% penicillin/streptomycin, 11.9 mM sodium bicarbonate, gentamicin (40 mg/mL), and 0.05 mM 2-mercaptoethanol. 0.5 × 10^5^ mAMs were pretreated with AGS for 24 h, exposed to 1 ng of EcoHIV overnight, and then AGS was reintroduced for an additional 48 h. The cells were gently washed once with media to remove membrane-bound or extracellular EcoHIV from the experimental system. At the end of the experiment, the media was removed and cells were washed with 1X phosphate-buffered saline (PBS) followed by fixing with 4% paraformaldehyde (PFA).

#### 2.5.2. MH-S Cells and THP-1-Derived Macrophages

The number of primary mAMs isolated from each mouse ranged between 0.2–0.3 × 10^6^ cells. Limitations in the number of isolated primary mAMs necessitated that some experiments be conducted by immunofluorescence and enzyme-linked immunosorbent assay (ELISA). Therefore, we utilized the MH-S cells, an AM cell line, for assays that required a greater number of cells. MH-S cells were initially purchased from the American Type Culture Collection (Manassas, VA, USA). MH-S cells were grown in RPMI-1640 medium containing 10% FBS, 1% penicillin/streptomycin, 11.9 mM sodium bicarbonate, gentamicin (40 mg/mL), and 0.05 mM 2-mercaptoethanol. Like primary mAMs, MH-S cells are permissive to EcoHIV and support EcoHIV’s replication as previously reported [18]. MH-S cells were pretreated with AGS for 24 h. After that, AGS was withdrawn, and 20 ng EcoHIV/1 × 10^6^ MH-S cells was introduced overnight. Extracellular EcoHIV was removed by washing the cells multiple times with media after overnight exposure, and AGS was then reintroduced for an additional 48 h. In another experiment, human monocyte leukemic cell lines, THP1 cells (ATCC, Manassas, VA, USA), were differentiated into macrophages by incubation with 10 ng/mL phorbol 12-myristate 13-acetate (PMA) for 24 h. After differentiation, THP1-derived macrophages were exposed to ±AGS for 24 h, followed by overnight treatment with BALF from PWH expressing the p24 antigen. The control cells were treated with BALF from PWoH to control BALF effects. The culture media containing BALF from PWH or PWoH were removed, and THP1-derived macrophages were washed with complete RPMI-1640 media to remove the residual BALF. THP1-derived macrophages were re-exposed to AGS for another 24 h. The cells were then harvested for assay.

### 2.6. Extracellular Flux Assays

Mitochondrial respiration was measured using the XFe96 Extracellular Flux Analyzer (Agilent Seahorse Bioscience Inc., Billerica, MA, USA) on a 96-well plate seeded with 0.2 × 10^5^ MH-S cells. Mitochondrial stress test assays were conducted to determine mitochondrial bioenergetics by measuring the oxygen consumption rate (OCR) of cells in response to 2 µM of oligomycin (ATP synthase inhibitor), 0.5 µM of carbonilcyanide p-triflouromethoxyphenylhydrazone, FCCP, (a protonophore), and 0.5 µM of rotenone (NADH dehydrogenase inhibitor) plus antimycin A (ubiquinol-cytochrome c reductase inhibitor). Before these compounds were injected into the cells, basal respiration, which is the amount of oxygen consumed by the cells to meet cellular energy demand, was evaluated. Then, the ATP-linked respiration, which measures the oxygen consumed to generate ATP needed to meet the energy demand of the cell, was determined by injecting the cells with oligomycin. ATP-linked respiration was derived from the difference between basal respiration and proton leak, a portion of basal respiration uncoupled for ATP production. Furthermore, the injection of FCCP collapsed the proton gradient by uncoupling protons from the inner mitochondrial membrane, consequently driving the cells to maximal respiration. The difference between maximal cellular respiration and basal respiration yielded spare respiratory capacity, which measures the cell’s ability to respond to energy demand in the presence of stressors. To account for non-mitochondrial oxygen consumption, which occurs during high ROS generation, antimycin A and rotenone injections were used to shut down the electron transport chain. The OCR was normalized to MH-S cell protein concentration (µg/µL). The OCR profiles were evaluated using XF Wave 2.1 software.

### 2.7. RNA Isolation, Real-Time Polymerase Chain Reaction

The TRIzol method was used to isolate total RNA [45] from which the mRNA levels of the EcoHIV constructs (*murine leukemia virus* (*MLV*) *enveloping protein*, *gag*, *Nef*, *Tat*, and *Vif*) were quantified by a quantitative real-time (qRT) polymerase chain reaction (PCR) with the sequences in Table 1. The iTaq Universal SYBR Green One-Step kit was used to measure target genes, which were normalized to glyceraldehyde-3-phosphate dehydrogenase (GAPDH).

### 2.8. Immunoblotting

Immunoblotting was performed as previously described [42]. Each gel lane was loaded with equal amounts (20 µg) of protein, as determined by the bicinchoninic acid assay. GAPDH was used as the loading control to normalize target proteins during band quantification. The Odyssey infrared imaging system (Li-Cor Bioscience, Lincoln, NE, USA) was used to develop blots. The densitometry of the protein bands was quantified using the National Institutes of Health (NIH) Image J software 1.5.4.

### 2.9. Immunofluorescence

The primary mAMs (0.5 × 10^5^/well) were seeded in 16-well Chambers culture slides. Cells were treated with AGS for 24 h, EcoHIV overnight, and then with AGS for an additional 48 h, as described in Section 2.5. At the end of the culture period, cells were washed once with 1× phosphate-buffered saline (PBS) and fixed with 4% PFA for 12 min at room temperature. Fixed cells were then permeabilized with 0.1% Triton X-100 in PBS for 3 min at room temperature and blocked for 30 min with 2% (*w*/*vol*) bovine serum albumin (BSA) in PBS. The cells were then incubated with the primary antibodies for 1 h at room temperature. The primary antibodies were then washed from the cells with 1X PBS. To visualize the primary antibodies, which had already bound to the target proteins, the cells were incubated for 30 min with secondary antibodies (Alexa Fluor 488 and Alexa Fluor 555). 4′,6-diamidino-2-phenylindole, DAPI, (Cat #62248, ThermoFisher Scientific, Waltham, MA, USA). DAPI was used to stain the cell nuclei. Some images were captured with a 40X lens on the Keyence BZ-X810 fluorescence microscope (Keyence, Itasca, IL, USA) and others with a 20X lens on the Nikon confocal microscope (Nikon, Melville, NY, USA).

### 2.10. Enzyme-Linked Immunosorbent Assay

We evaluated EcoHIV and HIV replication by measuring the release of p24 in culture media and BALF using HIV-1 gag p24 DuoSet ELISA (Cat. DY7360, R&D Systems, Minneapolis, MN, USA) according to the manufacturer’s protocol. Likewise, IL-1β released into the cell culture supernatant was measured using the Mouse IL-1 beta/IL-1F2 DuoSet ELISA (Cat. DY401, R&D Systems, Minneapolis, MN, USA).

### 2.11. Statistics

Data were analyzed using GraphPad Prism v10.0.3 software (GraphPad, La Jolla, CA, USA). Data from at least five duplicate independent experiments were expressed as mean ± SEM. Comparisons among multiple groups were performed using Analysis of Variance (ANOVA) and Tukey’s post hoc test. For comparisons between 2 groups, we used students’ *t*-tests. A *p*-value of 0.05 or less was considered significant.

## 3. Results

### 3.1. The Alveoli of PWH on ART Harbor Replication-Competent HIV, Which Is Potentiated by AGS

**Figure 1:**
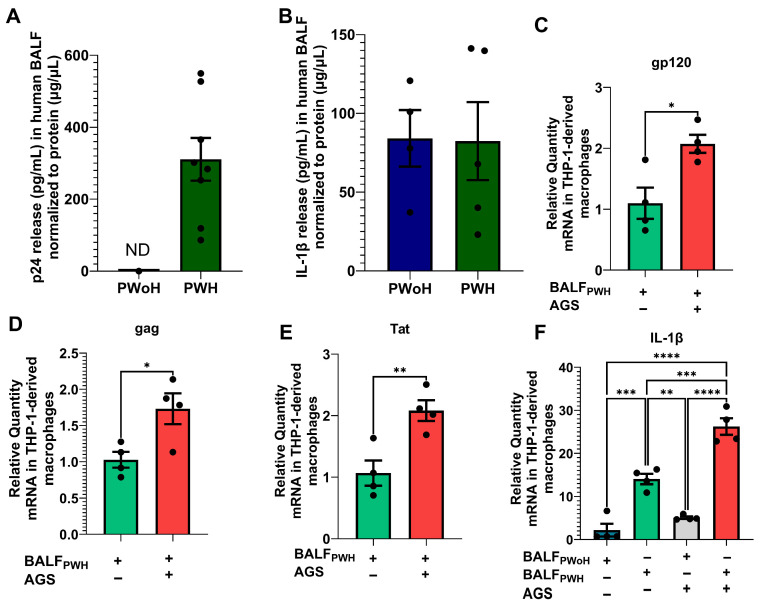
Detection of p24 antigen in the bronchoalveolar lavage fluid (BALF) from adult participants living with HIV (PWH) on ART compared with people without HIV (PWoH). (**A**) p24 antigen and (**B**) IL-1β release into the BALF of PWoH (*n* = 10) versus PWH (*n* = 7) was determined by ELISA. (**C**) gp120 (*n* = 4), (**D**) Gag (*n* = 4), (**E**) Tat (*n* = 4), and (**F**) IL-1β (*n* = 4) mRNA levels were measured by RT-PCR with GAPDH mRNA as the internal standard. All values are presented as the mean ± SEM. ND means not detected. * *p* < 0.05; ** *p* < 0.01; *** *p* < 0.001; **** *p* < 0.0001.

ART notwithstanding, several anatomical sites, including AMs, still harbor latent HIV proviruses [12,13,46]. However, it remains unclear whether replication-competent HIV exists in the alveoli. Therefore, we first examined the presence of HIV structural protein p24 in the BALF of 16 PWH who have been on ART for at least 18 months. In this analysis, BALF from 10 PWoH were included as negative controls. Since detection of p24 in acellular or extracellular environments may likely indicate HIV replication in surrounding cells, we used ELISA to detect p24 antigen in participants’ BALF. Our findings showed no detectable BALF p24 antigen in 50% (*n* = 8) of PWH. Conversely, the remaining 50% (*n* = 8) expressed p24 antigen, with levels ranging from 86 to 549 pg/mL, normalized to BALF protein concentration (µg/µL) (Figure 1A). Because HIV is known to activate proinflammatory responses in AMs, we also used ELISA to examine IL-1β levels in the BALF. IL-1β was detected in 31% of PWH compared to 40% of PWoH, but there was no statistically significant difference in BALF IL-1β levels between the two groups (Figure 1B). To assess whether p24 detected in BALF of 50% of PWH represented replication-competent HIV particles when exposed to alcohol metabolism, we pooled BALF from the PWH who expressed p24 antigen. Then, AGS-exposed THP1–derived macrophages were treated with 10 pg of p24 from the BALF of PWH. After 24 h, AGS upregulated mRNA levels for *gp120* (Figure 1C), *gag* (Figure 1D), and *Tat* (Figure 1E) in THP1-derived macrophages. Furthermore, we found increased *IL-1β* mRNA levels in THP1-derived macrophages exposed to BALF from PWH, and this effect was enhanced by AGS (Figure 1F).

### 3.2. AGS Potentiates EcoHIV Expression and IL-1β Activation in Primary Mouse AMs

**Figure 2:**
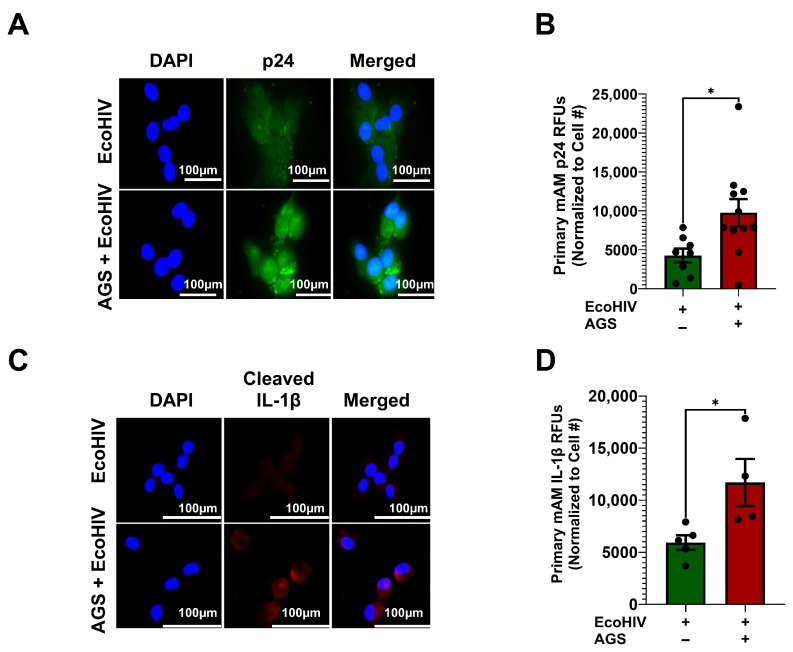
Acetaldehyde generating system (AGS) enhances p24 antigen and cleaved interleukin (IL)-1β expression in EcoHIV-infected primary mouse alveolar macrophages (mAMs). 0.5 × 10^5^ primary mAMs seeded on Chamber’s culture slides were exposed ± AGS for 24 h, infected ± 1 ng of EcoHIV overnight, and then ± AGS for an additional 48 h. (**A**–**D**) p24 antigen (*n* = 8–10) and cleaved IL-1β (*n* = 4) staining of primary mAMs and quantification of the immunostaining intensity using NIH ImageJ 1.5.4. as relative fluorescence intensity (RFU). Fluorescence intensity was normalized to cell numbers. Staining was visualized with the 40X lens of the Keyence BZ-X810 fluorescence microscope. Data are presented as the mean ± SEM. A two-sample independent *t*-test was used to compare the groups. * *p* < 0.05.

Previously, we showed that AGS enhances HIV replication in AMs, as indicated by the release of p24 antigen into the extracellular environment [18]. However, the ability of acetaldehyde to promote HIV protein retention in AMs during replication was not defined. Therefore, we evaluated intracellular expression of p24 antigens in AMs to examine whether AGS enhances EcoHIV protein accumulation in primary mAMs. AGS doubled p24 expression in EcoHIV-infected primary mAMs (Figure 2A,B). Intracellular accumulation of p24 proteins acts as a viral pathogen-associated molecular pattern (PAMP), which triggers chronic inflammatory responses in tissue reservoirs [47]. Therefore, we investigated whether the accumulation of p24 proteins by AGS triggers IL-1β activation in AMs. As anticipated, we found that AGS increased IL-1β activation (as demonstrated by cleavage) in EcoHIV-infected primary mAMs (Figure 2C,D).

### 3.3. AGS Enhances Mitochondrial Bioenergetics in EcoHIV-Infected MH-S Cells

**Figure 3:**
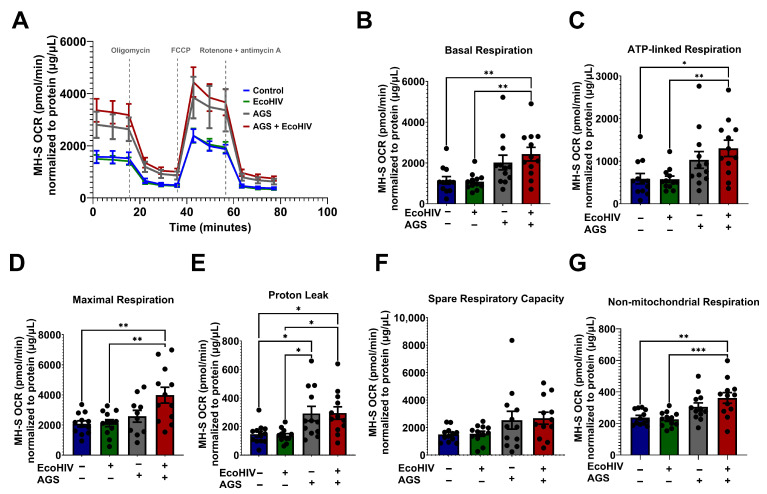
Acetaldehyde generating system (AGS) enhanced the oxygen consumption rate in EcoHIV-infected MH-S cells. 0.2 × 10^5^ MH-S cells seeded on XFe96 cell culture microplates were exposed ± AGS for 24 h. Then, the cells were infected ± 400 pg of EcoHIV overnight. Extracellular EcoHIV was then gently washed off with serum-free medium. AGS was reintroduced or not for an additional 48 h. The oxygen consumption rate (OCR) of cells at baseline and after a series of mitochondrial function-related injections using a mitochondrial stress test (oligomycin, carbonylcyanide p-trifluoromethoxyphenylhydrazone, FCCP, and rotenone/antimycin) was measured using the Agilent Seahorse XFe96 extracellular flux analyzer. (**A**) The average OCR bioenergetic profiles of MH-S cells (*n* = 12). OCRs are shown for (**B**) basal respiration (*n* = 12), (**C**) ATP-linked respiration (*n* = 12), (**D**) maximal respiration (*n* = 12), (**E**) proton leak (*n* = 12), (**F**) spare respiratory capacity, (*n* = 12), and (**G**) non-mitochondrial respiration (*n* = 12). OCR measurements were normalized to protein content (µg/µL) in the same sample. Data are presented as means ± SEM. Comparisons among multiple groups were performed using ANOVA with Tukey’s post hoc test. * *p* < 0.05; ** *p* < 0.01; *** *p* < 0.001.

It has been reported that HIV alters glucose uptake in various immune cells [48]. Additionally, we recently demonstrated that chronic alcohol exposure results in mitochondrial disruption and altered glycolysis in AMs [25,35,49]. However, a knowledge gap remains regarding the direct effects of acetaldehyde on the mitochondria of HIV-infected AMs. This is an important question as acetaldehyde detoxification occurs in mitochondria. Acetaldehyde is also a substrate for acetate, which is a key component of the Krebs cycle. It has been shown that acetate increases mitochondrial respiration in different cell models [50,51,52]. Therefore, we examined whether acetaldehyde enhances mitochondrial respiration in HIV-infected AMs. AGS + EcoHIV significantly enhanced OCR bioenergetic profiling (Figure 3A) and basal respiration (Figure 3B) compared with the EcoHIV and control groups. In comparison to MH-S cells not exposed to AGS, the combination of AGS and EcoHIV concurrently enhanced ATP-linked respiration (Figure 3C), maximal respiration (Figure 3D), proton leak (Figure 3E), spare respiratory capacity (Figure 3F), and non-mitochondrial respiration (Figure 3G).

### 3.4. AGS Increases Mitochondrial ATP Production and Mitochondrial ROS in MH-S Cells

**Figure 4:**
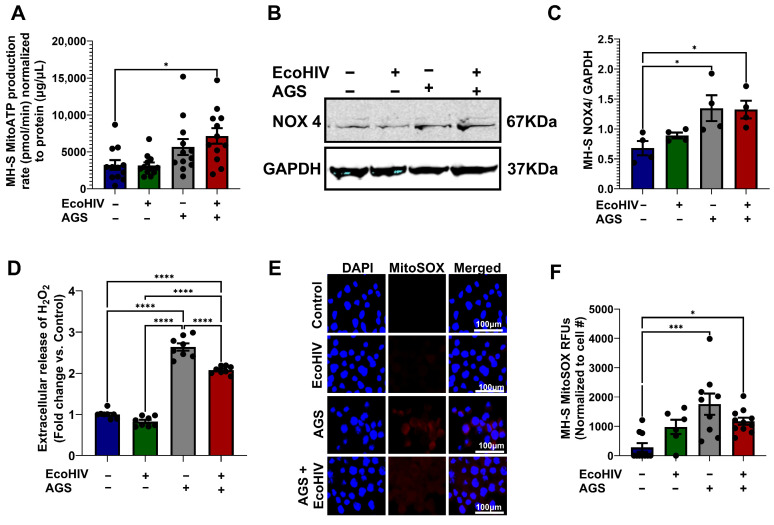
Acetaldehyde generating system (AGS) promotes ATP and reactive oxygen species (ROS) production in MH-S cells. MH-S cells were pretreated with AGS for 24 h. After that, AGS was removed, and 20 ng of EcoHIV per 1 × 10^6^ MH-S cells was added for 16 h or overnight. Extracellular EcoHIV was then gently washed off with serum-free medium. AGS was reintroduced or not for an additional 48 h. (**A**) Mitochondrial ATP production rate was measured by computing ATP-linked respiration with the number of ATP generated per oxygen atom (*n* = 11–12). (**B**,**C**) Immunoreactive bands obtained in Western Blot analysis of NOX4 (*n* = 4). Bands were quantified and normalized to GAPDH. Western blot original images can be found in Supplementary Materials. (**D**) Amplex Red assay measurement of H_2_O_2_ released by MH-S cells in culture supernatants (*n* = 4). (**E**,**F**) MitoSOX (*n* = 7) staining of MH-S cells and quantification of the immunostaining intensity using NIH ImageJ 1.5.4. as relative fluorescence intensity (RFU). Comparisons among multiple groups were performed using ANOVA with Tukey’s post hoc test. * *p* < 0.05; *** *p* < 0.001; **** *p* < 0.0001.

Since mitochondrial hyperactivity is characterized by increased mitochondrial ATP production and ROS generation, we examined whether AGS-induced oxygen consumption elevates mitochondrial ATP production and ROS in EcoHIV-infected MH-S cells. It is important to note that reduced flavine adenine dinucleotide (FADH2) and NADH are the primary electron donors responsible for driving mitochondrial ATP production. The culture medium used for MH-S cells, RPMI, does not contain succinate or other precursors that promote FADH2-driven ATP production. However, RPMI does contain nicotinamide, which means that NADH serves as the main electron donor in MH-S cells. When NADH is the major electron donor, the mitochondrial ATP produced per molecule of oxygen (O2) is approximately 2 × 2.75. Therefore, the mitochondrial ATP production rate for MH-S cells was calculated by multiplying the ATP-linked respiration by 2 × 2.75. AGS increased ATP production in EcoHIV-infected MH-S cells (Figure 4A). NOX4, which localizes to mitochondria, plays critical roles in oxidative stress responses. Therefore, we determined whether AGS increases NOX4 expression. We found that AGS, with or without EcoHIV infection, increased NOX4 expression (Figure 4B,C) and hydrogen peroxide (H_2_O_2_) production (Figure 4D), an ROS generated by NOX4. Furthermore, AGS concurrently increased mitochondrial superoxide (MitoSOX) in MH-S cells (Figure 4E,F).

### 3.5. Pyruvate and Glutamine Fuel AGS-Induced Mitochondrial Respiration in EcoHIV-Infected MH-S Cells

**Figure 5:**
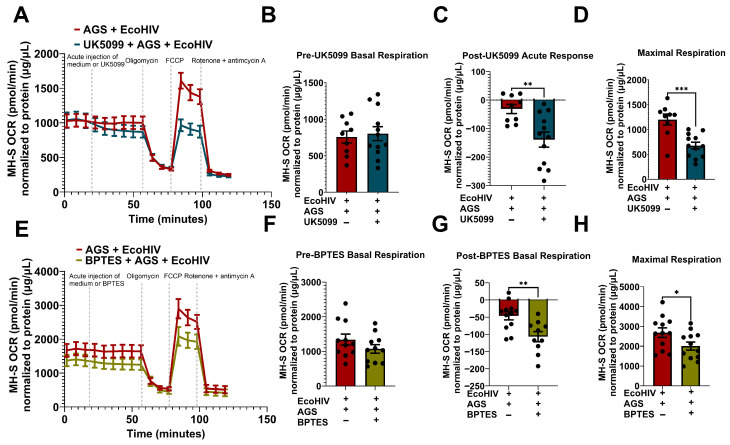
Acetaldehyde generating system (AGS) enhances mitochondrial bioenergetics through pyruvate and glutamine metabolism in EcoHIV-infected MH-S cells. 0.2 × 10^5^ MH-S cells seeded on XFe96 cell culture microplates were exposed ± AGS for 24 h, followed by infection ± 400 pg of EcoHIV for 24 h. Extracellular EcoHIV was then gently washed off with serum-free medium, and cells were treated ± AGS for 48 h. The Agilent Seahorse XFe96 extracellular flux analyzer measured the OCR of MH-S cells at baseline and after injection with mitochondrial pyruvate carrier inhibitor, 2 µM UK5099, or glutaminolysis inhibitor, 3 µM BPTES. (**A**) The average OCR bioenergetic profiles of MH-S cells (*n* = 12) during a pyruvate oxidation stress test. The OCRs for this test are shown in (**B**) pre-UK5099 basal respiration (*n* = 12), (**C**) post-UK5099 basal respiration (*n* = 12), and (**D**) maximal respiration (*n* = 12). (**E**) The average OCR bioenergetic profiles of MH-S cells (*n* = 11) during a glutamine oxidation stress test. The OCRs for this test are shown in (**F**) pre-BPTES basal respiration (*n* = 11), (**G**) post-BPTES basal respiration (*n* = 11), and (**H**) maximal respiration (*n* = 11). OCR measurements were normalized to protein content (µg/µL) in the same sample. Data are expressed as the mean ± SEM. A two-sample independent t-test was used to compare the groups. * *p* < 0.05; ** *p* < 0.01; *** *p* < 0.001.

To determine whether pyruvate and glutamine were being utilized by the cells for mitochondrial respiration when infected with EcoHIV and exposed to AGS, the cells were injected with UK5099 (a mitochondrial pyruvate carrier inhibitor) and BPTES (an inhibitor of glutaminolysis). UK5099 decreased OCR in AGS + EcoHIV-infected MH-S cells (Figure 5A). As expected, there was no difference between the basal respiration of cells designated to receive media versus UK5099 injections (Figure 5B). However, we observed an acute response to UK5099 in EcoHIV-infected MH-S cells + AGS (Figure 5C), which also attenuated the maximal respiration (Figure 5D) in these cells. BPTES decreased the OCR bioenergetic profile of AGS + EcoHIV-infected MH-S cells (Figure 5E). While there were no changes in the basal respiration of EcoHIV-infected MH-S cells + AGS before BPTES injection (Figure 5F), we observed an acute response to BPTES after injection (Figure 5G). Furthermore, there was a difference in maximal respiration between AGS + EcoHIV MH-S cells injected with BPTES and those injected with media (Figure 5H). This suggests that, beyond the dependency of EcoHIV-infected MH-S cells exposed to AGS on pyruvate and glutamine for mitochondrial respiration, they also require these substrates to attain the highest oxidative capacity.

### 3.6. AGS-Induced Mitochondrial Respiration Supports EcoHIV Replication and IL-1β Activation in Primary mAMs and MH-S Cells

**Figure 6:**
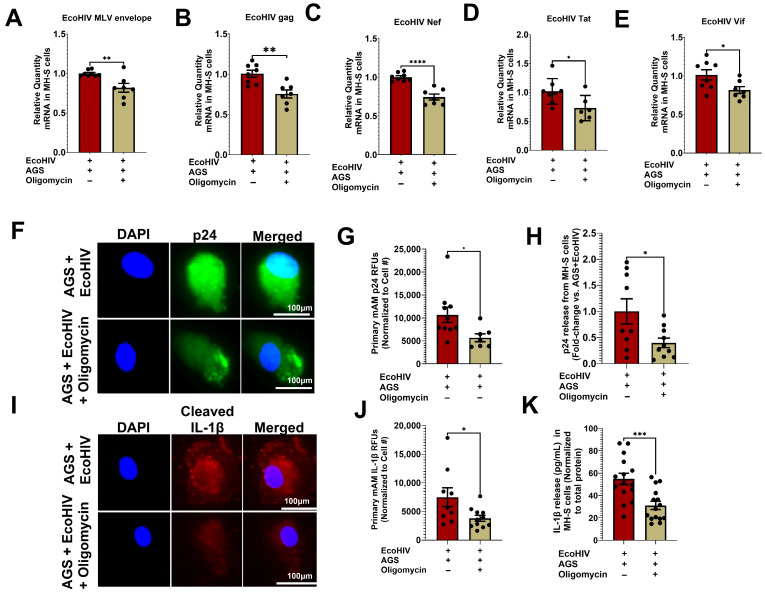
Inhibition of mitochondrial ATP synthesis suppresses acetaldehyde-generating system (AGS)-induced mitochondrial bioenergetics in EcoHIV-infected AMs. Primary AMs and MH-S cells were pretreated ± AGS for 24 h, followed by ± 1 ng of EcoHIV per 0.5 × 10^5^ cells for 2 h. Then, the cells were treated with 1 µM oligomycin and immediately withdrawn after 1 h. 0.3 µM oligomycin was then reapplied or not with AGS for an additional 48 h. EcoHIV mRNA levels of (**A**) MLV envelope protein (*n* = 5), (**B**) Gag (*n* = 7), (**C**) Nef (*n* = 6), (**D**) Tat (*n* = 6), and (**E**) Vif (*n* = 7) in MH-S cells were measured by qRT-PCR, normalized to GAPDH, and expressed relative to EcoHIV-infected cells exposed to AGS. (**F**,**G**) p24 (*n* = 7) staining of primary mAMs and quantification of the immunostaining intensity using NIH ImageJ 1.5.4. as relative fluorescence intensity (RFU). Fluorescence intensity was normalized to cell numbers. Staining was visualized with the 40X lens of the Keyence BZ-X810 fluorescence microscope (**H**) p24 release (*n* = 9) from primary mAMs was measured in the culture supernatant by ELISA and normalized to total cell protein. Values are expressed as fold change of AGS + EcoHIV + Oligomycin compared to AGS + EcoHIV (**I**,**J**) Cleaved IL-1β (*n* = 9) staining of primary mAMs and quantification of the immunostaining intensity using NIH ImageJ 1.5.4. as relative fluorescence intensity (RFU). Fluorescence intensity was normalized to cell numbers. Staining was visualized with the 40X lens of the Keyence BZ-X810 fluorescence microscope. (**K**) IL-1β release (*n* = 13) from primary mAMs was measured in the culture supernatant by ELISA and normalized to total cell protein. Data are expressed as the mean ± SEM. A two-sample independent *t*-test was used to compare the groups. * *p* < 0.05; ** *p* < 0.01; *** *p* < 0.001; **** *p* < 0.0001.

AGS enhanced HIV replication and IL-β activation, as demonstrated in MH-S cells [18] and in primary mAMs (Figure 2). AGS also promoted mitochondrial bioenergetics profiling in EcoHIV-infected AMs (Figure 3), remodeling them into a mitochondrial hyperactive phenotype (Figure 4) via pyruvate and glutamine metabolism (Figure 5). Since a mitochondrial hyperactive phenotype describes a compensatory mechanism to meet the energy demand of an infection or immune response [53,54,55], we investigated whether AGS-induced mitochondrial ATP synthesis supports HIV replication and IL-1β release in AMs. AGS-exposed EcoHIV-infected AMs were treated with 1 µM oligomycin for 1 h, followed by chronic treatment with 0.3 µM oligomycin for 48 h. Treatment of the cells with 1 µM oligomycin for 1 h sufficiently inhibits ATP production before apoptosis is triggered. Since it required at least 48 h to observe a significant replication of EcoHIV in the cells [18], the oligomycin dose was decreased to 0.3 µM oligomycin, which was sufficient to continue to impair mitochondrial respiration without triggering significant apoptosis [56]. In AGS-exposed EcoHIV-infected MH-S cells, oligomycin attenuated EcoHIV constructs such as mRNA for *MLV envelope protein* (Figure 6A), *Gag* (Figure 6B), *Nef* (Figure 6C), *Tat* (Figure 6D), and *Vif* (Figure 6E). Additionally, oligomycin attenuated p24 expression in AGS-exposed EcoHIV-infected primary mAMs (Figure 6F,G) and its release from MH-S cells (Figure 6H). Oligomycin also suppressed IL-1β activation in AGS-exposed EcoHIV-infected primary mAMs (Figure 6I,J) and its release from MH-S cells (Figure 6K).

### 3.7. Increased p65 Expression in Primary Human AMs (hAMs) from Individuals with AUD and AGS Drives p65 Activation in EcoHIV-Infected Primary mAMs

**Figure 7:**
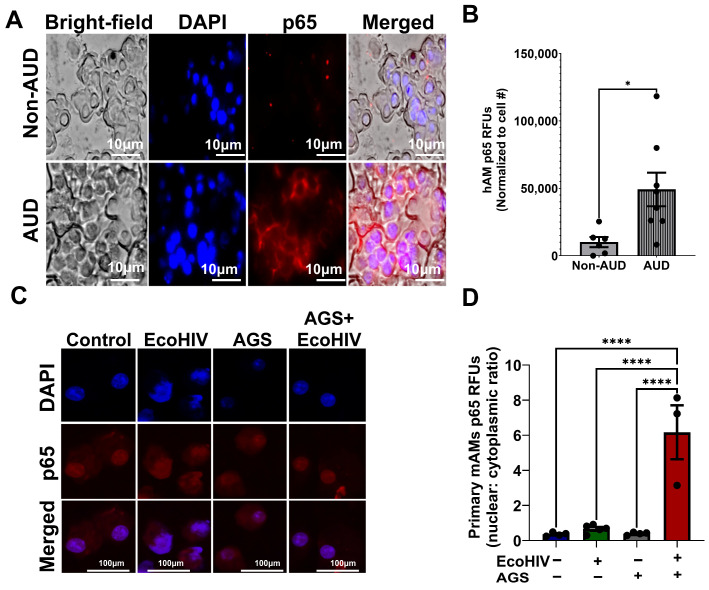
Nuclear factor kappa B p65 (p65) expression is increased in the primary alveolar macrophages (hAMs) of individuals with alcohol use disorder (AUD), and AGS activates p65 in primary mouse alveolar macrophages (mAMs). (**A**,**B**) hAMs from individuals with AUD and without AUD were immunostained with anti-p65 (*n* = 6–8). Immunofluorescence staining of p65 was visualized on a Keyence BZ-X810 fluorescence microscope using a 40X lens, and the fluorescence intensity was quantified using NIH ImageJ 1.5.4 as relative fluorescence intensity (RFU). Fluorescence intensity was normalized to cell number. (**C**,**D**) Active p65 was measured by the ratio of the fluorescence intensity of the nuclear p65 immunostaining versus the cytoplasmic p65 immunostaining of the cells (*n* = 3–5). Staining was visualized on a Nikon confocal microscope using a 20X lens. Fluorescence intensity was normalized to cell number. All values were presented as the mean ± SEM. Comparisons among multiple groups were performed using ANOVA with Tukey’s post hoc test. A two-sample independent *t*-test was used to compare the groups. * *p* < 0.05; **** *p* < 0.0001.

We previously showed that AGS increases p65 expression and promotes p65 activation in EcoHIV-infected MH-S cells. We also linked p65 activation to AGS-induced HIV replication and IL-1β release [18]. Here, we examined p65 expression in the hAMs of individuals with AUD. AMs were isolated from the BALF of individuals with and without AUD. As shown in Table 2, the participants averaged 39.78 years of age. None used marijuana, cocaine, or tobacco. Most participants were Black (79%), and half were male. Fifty-seven percent of the participants had AUD (Table 2). p65 expression was increased in the hAMs of individuals with AUD compared to those without AUD (Figure 7A,B), which supports our previous data in MH-S cells [18]. Additionally, to confirm that alcohol metabolism increases p65 activity during HIV infection, we exposed primary mAMs to EcoHIV and AGS. Similarly to our published results in EcoHIV-infected MH-S cells [18], AGS increased p65 activity in EcoHIV-infected primary mAMs as indicated by an increased p65 nuclear-cytoplasmic ratio (Figure 7C,D).

### 3.8. Inhibition of p65 Suppresses AGS-Induced Mitochondrial Bioenergetics in EcoHIV-Infected MH-S Cells

**Figure 8:**
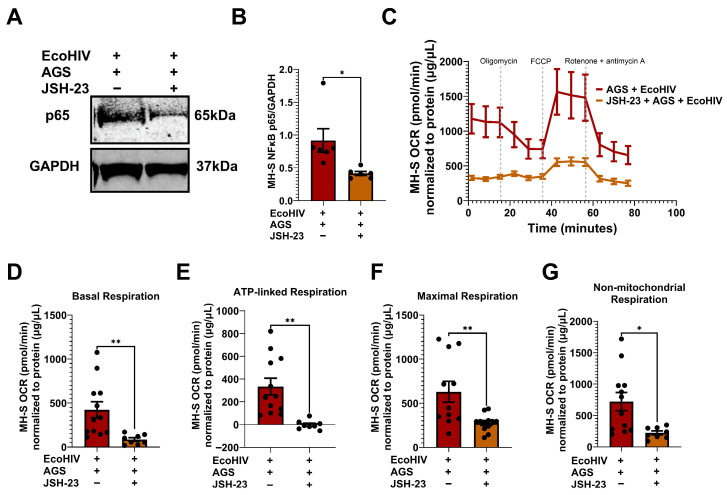
p65 promotes acetaldehyde generating system (AGS)-induced mitochondrial bioenergetics in EcoHIV-infected MH-S cells. MH-S cells were pretreated ± 50 μM JSH-23, a p65 activation inhibitor, for 24 h, followed by ± AGS for 24 h and infection ± EcoHIV for 24 h. The extracellular EcoHIV was removed by washing the cells several times with serum-free medium, and cells were treated ± 50 µM JSH-23 and ± AGS for 48 h. (**A**,**B**) The effect of JSH-23 on p65 expression in MH-S cells (*n* = 6) was assessed using immunoblot analysis, and protein bands were quantified and normalized to GAPDH. Western blot original images can be found in Supplementary Materials. (**C**) The average OCR bioenergetic profiles of MH-S cells (*n* = 12) during a mitochondrial stress test (*n* = 12). The OCRs for this test are shown for (**D**) basal respiration (*n* = 12), (**E**) ATP-linked respiration (*n* = 12), (**F**) maximal respiration (*n* = 12), and (**G**) non-mitochondrial respiration (*n* = 12). The OCR measurements were normalized to protein concentration (µg/µL) in the same sample. Data are presented as the mean ± SEM. A two-sample independent *t*-test was used to compare the groups. * *p* < 0.05; ** *p* < 0.01.

**Figure A1:**
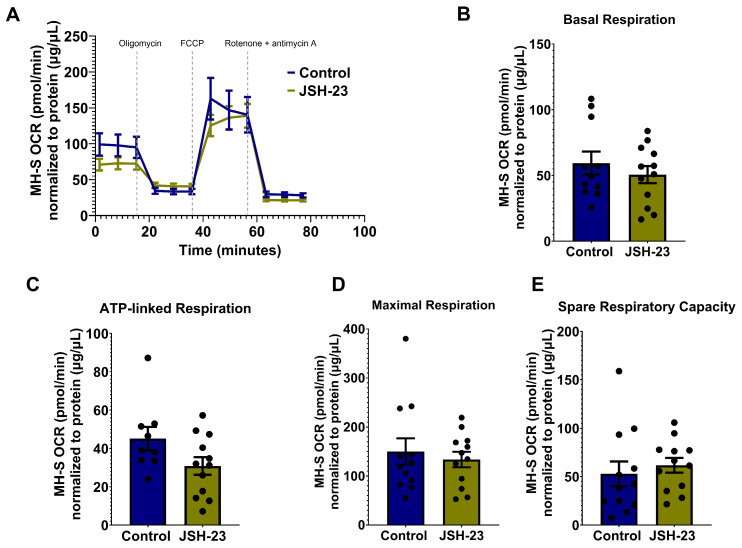
JSH-23, a p65 activation inhibitor, does not change mitochondrial bioenergetics in control MH-S cells. MH-S cells were treated ± with 50 μM JSH-23 for 48 h. (**A**) The average OCR bioenergetic profiles of MH-S cells (*n* = 12) during a mitochondrial stress test (*n* = 12). The OCRs for this test are shown for (**B**) basal respiration (*n* = 12), (**C**) ATP-linked respiration (*n* = 12), (**D**) maximal respiration (*n* = 12), and (**E**) spare respiratory capacity (*n* = 12). The OCR measurements were normalized to protein concentration (µg/µL) in the same sample. Data are presented as the mean ± SEM. A two-sample independent *t*-test was used to compare the groups, but none of the groups in the panels were significantly different at *p* < 0.05.

While it has been established that p65 drives AGS-induced HIV replication and IL-1β release, the mechanism underlying this relationship remains elusive. Given that p65 promotes stress-induced mitochondrial hyperactivity [22,57], which AGS also triggers in EcoHIV-infected AMs, we aimed to investigate whether AGS-induced p65 contributes to mitochondrial hyperactivity in AMs. EcoHIV-infected MH-S cells exposed to AGS were treated with a pharmacological inhibitor of p65 expression and activity, JSH-23. As indicated in Figure 8A,B, JSH-23 decreased p65 expression in EcoHIV-infected MH-S cells exposed to AGS. Furthermore, JSH-23 decreased OCR in AGS + EcoHIV MH-S cells (Figure 8C). Basal respiration (Figure 8D), ATP-linked respiration (Figure 8E), maximal respiration (Figure 8F), and non-mitochondrial respiration (Figure 8G) were attenuated by JSH-23 treatment of EcoHIV-infected MH-S cells exposed to AGS. These effects were not observed in control cells treated with JSH-23 (Figure A1).

### 3.9. PIO, Which Inhibits p65 Activation, Restores Mitochondrial Bioenergetics in EcoHIV-Infected MH-S Cells Exposed to AGS

**Figure 9:**
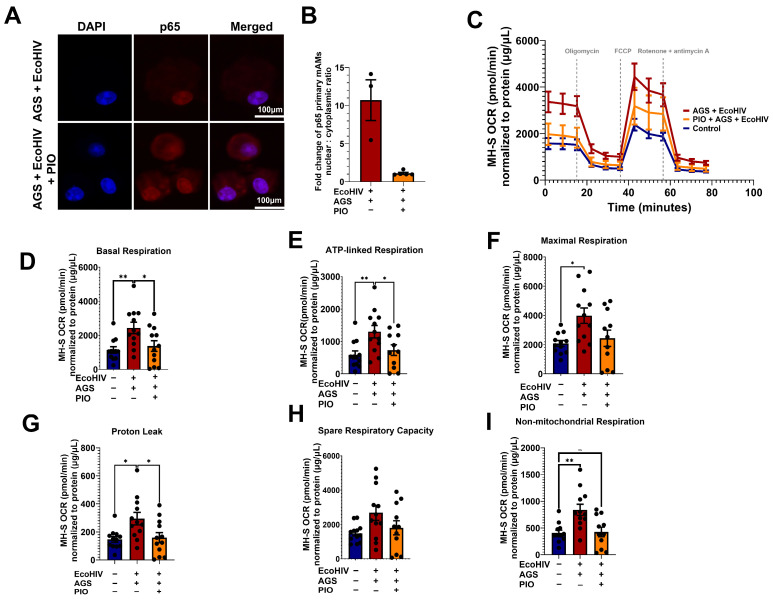
Pioglitazone (PIO) inhibits p65 activation and restores acetaldehyde generating system (AGS)-induced mitochondrial bioenergetics to control levels. Primary mAMs and MH-S cells were pretreated ± AGS for 24 h, followed by infection ± 400 pg of EcoHIV per 0.2 × 10^5^ cells for 24 h and ± AGS for 48 h. During the last 24 h of AGS treatment, 10 µM PIO was added or not as an intervention therapy. (**A**,**B**) p65 activation was measured by assessing the nuclear-to-cytoplasmic fluorescence intensity ratio of p65 immunostaining (*n* = 3–5). Fluorescence intensity was normalized to cell number. Staining was visualized on a Nikon confocal microscope using a 20X lens. (**C**) The average OCR bioenergetic profiles of MH-S cells (*n* = 12) during a mitochondrial stress test. The OCRs for this test are shown for (**D**) basal respiration (*n* = 12), (**E**) ATP-linked respiration (*n* = 12), (**F**) maximal respiration (*n* = 12), (**G**) proton leak (*n* = 12), (**H**) spare respiratory capacity (*n* = 12), and (**I**) non-mitochondrial respiration (*n* = 12). The OCR measurements were normalized to protein concentration (µg/µL) in the same sample. Data are presented as the mean ± SEM. Comparisons among multiple groups were performed using ANOVA with Tukey’s post hoc test. A two-sample independent *t*-test was used to compare the groups. * *p* < 0.05; ** *p* < 0.01; ns = non-significant.

**Figure A2:**
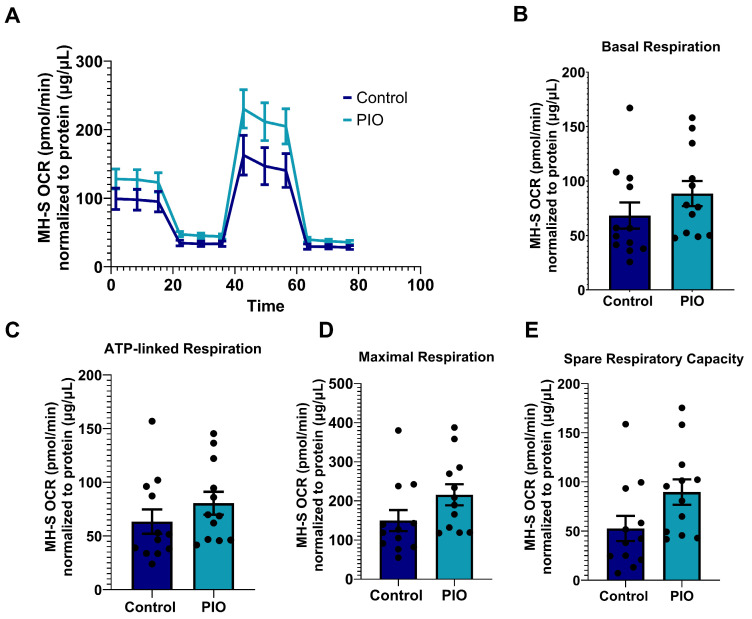
Pioglitazone (PIO) does not change mitochondrial bioenergetics in control MH-S cells. MH-S cells were treated ± with 10 μM PIO for 24 h. (**A**) The average OCR bioenergetic profiles of MH-S cells (*n* = 12) during a mitochondrial stress test. The OCRs for this test are shown for (**B**) basal respiration (*n* = 12), (**C**) ATP-linked respiration (*n* = 12), (**D**) maximal respiration (*n* = 12), and (**E**) spare respiratory capacity (*n* = 12). The OCR measurements were normalized to protein concentration (µg/µL) in the same sample. Data are presented as the mean ± SEM. A two-sample independent *t*-test was used to compare the groups, but none of the groups in the panels were significantly different at *p* < 0.05.

Recently, we showed that treatment with PIO enhances the expression of PPARγ, a negative regulator of p65, in EcoHIV-infected primary mAMs exposed to AGS [18]. Here, we confirmed that PIO decreased AGS + EcoHIV-induced p65 activity in primary mAMs (Figure 9A,B). Since AGS-induced p65 is a regulator of mitochondrial bioenergetics in EcoHIV-infected MH-S cells (Figure 8), we expect inhibition of p65 activity with PIO treatment will restore mitochondrial bioenergetic function. 10 µM PIO treatment, which attenuated HIV replication and IL-1β activation [18], decreased OCR in EcoHIV-infected AGS-exposed MH-S cells to levels similar to those of the control group (Figure 9C). Furthermore, PIO attenuated the basal respiration (Figure 9D), ATP-linked respiration (Figure 9E), maximal respiration (Figure 9F), proton leak (Figure 9G), spare respiratory capacity (Figure 9H), and non-mitochondrial respiration (Figure 9I). However, PIO did not have a significant effect on the control cells (Figure A2).

### 3.10. PIO Modulates Alcohol Metabolism-Induced ATP and ROS Production in MH-S Cells

**Figure 10:**
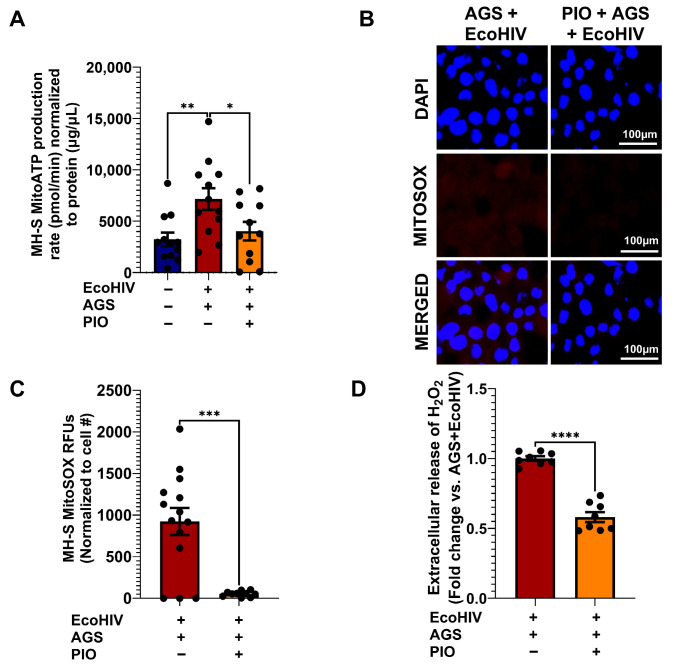
PIO attenuates Acetaldehyde-generating system (AGS)-induced ATP and reactive oxygen species (ROS) production in EcoHIV-infected MH-S cells. MH-S cells were pretreated with AGS for 24 h, then infected with 20 ng of EcoHIV per 10^6^ cells for 24 h, and treated with AGS for another 48 h. During the final 24 h of AGS treatment, 10 µM PIO was added or not, as a form of intervention therapy. (**A**) Mitochondrial ATP production rate in MH-S cells was measured by computing ATP-linked respiration with the number of ATP generated per oxygen atom (*n* = 10). (**B**,**C**) Immunofluorescence staining of MitoSOX (*n* = 13) was visualized on a Keyence BZ-X810 fluorescence microscope using a 40X lens, staining of MH-S cells and quantification of the immunostaining intensity using NIH ImageJ 1.5.4. as relative fluorescence intensity (RFU). (**D**) Amplex Red assay measurement of H_2_O_2_ released by MH-S cells in culture supernatants (*n* = 8). A two-sample independent *t*-test was used to compare the two groups. Comparisons among multiple groups were performed using ANOVA with Tukey’s post hoc test. * *p* < 0.05; ** *p* < 0.01; *** *p* < 0.001; **** *p* < 0.0001.

It is clear from Figure 3, Figure 4 and Figure 8 that AGS-induced p65 activation leads to mitochondrial hyperactivity. Therefore, we examined whether PIO can reverse the mitochondrial hyperactive phenotype in AGS-exposed EcoHIV-infected AMs. PIO restored mitochondrial ATP production to levels similar to control cells (Figure 10A) and also decreased AGS-induced MitoSOX production (Figure 10B,C) and H_2_O_2_ (Figure 10D) in EcoHIV-infected MH-S cells.

## 4. Discussion

This study detected replication-competent HIV in the alveoli of some PWH despite these same individuals having an elevated CD4+ cell count and an undetectable plasma HIV load. This is critical since HIV in the alveoli, even with ART, is associated with impaired lung immune functions [12], which increases the risk of lung infections, injury, and fibrosis. Although HIV drives pulmonary illnesses, alcohol misuse, which is frequently observed among PWH, exacerbates this risk. We recently demonstrated that acetaldehyde activates p65, leading to HIV replication and IL-1β activation in AMs. Furthermore, we showed that PIO, a negative regulator of p65, suppresses HV replication and IL-1β activation in AMs [18]. Here, we demonstrated that acetaldehyde-induced p65 activation promotes mitochondrial hyperactivation as an adaptive response for HIV replication and IL-1β responses in AMs. Furthermore, treatment with PIO suppresses acetaldehyde and HIV-induced p65 activation and restores mitochondrial bioenergetics to control levels, thereby attenuating HIV replication and IL-1β release in AMs.

Tremendous efforts have been directed towards ending the AIDS pandemic by 2030 [58]. However, this goal is severely challenged by various factors, including the persistence of HIV reservoirs in ART-experienced PWH. Although HIV in reservoirs was once thought to be transcriptionally silent, with advances in laboratory technology, low-level HIV replication has been demonstrated in some reservoirs of ART-experienced PWH [15,59]. This suggests that ART penetration into these anatomical sites may be suboptimal. Therefore, developing therapeutic strategies with optimal potency in these anatomical sites may move us one step closer to ending the AIDS pandemic, especially among PWH who misuse alcohol [60]. Treating PWH who misuse alcohol is essential since alcohol misuse fosters non-adherence to treatment [61], enhances viremia [62], and attenuates immune response [63], resulting in worse HIV outcomes [64]. Alcohol metabolism triggers HIV replication and IL-1β activation in AMs, so it is feared that this may become a source of HIV rebound, maintenance of HIV reservoirs, and chronic inflammation in the lungs when ART is interrupted, which is common among PWH who misuse alcohol. To provide adequate intervention for PWH who misuse alcohol, we must first uncover the mechanisms by which acetaldehyde triggers HIV replication and proinflammatory responses in AMs.

HIV exploits the same mitochondrial bioenergetic pathway that is canonically used for cellular anabolic processes to replicate and induce inflammatory responses [65]. In contrast, mitochondrial dysfunction in AMs has been attributed to alcohol exposure and HIV infection [35,66]; however, we unveiled a novel insight that both alcohol metabolism and EcoHIV enhanced mitochondrial bioenergetics in AMs, more so than either stress alone, thus demonstrating that the effects of alcohol metabolism and HIV on AM’s mitochondrial respiration are synergistic. Additionally, inhibition of mitochondrial ATP synthesis with low-dose oligomycin attenuated HIV replication and IL-1β activation, suggesting that mitochondrial hyperactivity in AMs is an adaptive response to the energy demands imposed by HIV replication and acetaldehyde-induced proinflammatory activation. We also showed that acetaldehyde-induced mitochondrial respiration depends on pyruvate and glutamine metabolism for HIV replication and proinflammatory changes in AMs, as evidenced by the decrease in OCR observed with UK5099 and BPTES. While the underlying mechanism for this phenomenon remains unclear, we were able to reverse acetaldehyde-induced mitochondrial hyperactivity by inhibiting the p65 signaling pathway with JSH-23. Therefore, p65 contributes to alcohol metabolism-induced mitochondrial hyperactive state in HIV-infected AMs. We previously observed in AMs that increased ATP-linked respiration was beneficial for AMs’ immune functions [25], especially when the OCR of non-mitochondrial respiration and proton leak is concurrently low. However, this is not the case for HIV-infected AMs exposed to AGS. ATP-linked respiration, proton leak, and non-mitochondrial respiration were increased in HIV-infected AMs exposed to acetaldehyde. Elevated proton leak and non-mitochondrial respiration suggest that AMs are exerting more effort to meet their ATP demand, as validated by elevated maximum respiration with a discordant spare respiratory capacity. This potentially leads to increased oxidative stress, as we have seen previously [18], and mitochondrial dysfunction in HIV-infected AMs exposed to acetaldehyde.

Alcohol metabolism-induced ROS production in AMs is well described [25,67,68,69]. However, it is unclear what the specific roles of HIV infection are as a second hit to the oxidative response of acetaldehyde-exposed AMs. In this study, we demonstrated that AGS increased NOX4 expression and ROS production, and EcoHIV, which did not alter AGS-induced NOX4 expression, attenuated ROS production in MH-S cells (Figure 4). Interestingly, EcoHIV, while not affecting AGS-induced NOX4 expression, reduced ROS production in these cells. Although ROS levels in EcoHIV-exposed MH-S cells remained significantly higher than in cells that were not exposed to either AGS or EcoHIV, these findings offer insights into the redox biology of AMs in the context of alcohol metabolism and HIV infection. It was previously reported that elevated oxidative stress during HIV infection stimulates the endogenous antioxidant pathways [70], which may temporarily offer a protective response from oxidative stress. This may explain the protective effects of EcoHIV infection to AGS-induced oxidative stress in AMs. However, prolonged activation of these pathways can eventually lead to exhaustion of endogenous antioxidants. Consequently, the cells may shift from a phase of antioxidant response to a state of overt oxidative stress [26,71,72].

Previously we demonstrated in murine AMs that acetaldehyde increased total p65 expression [18]. Here, we replicated this effect in human AMs when increased p65 expression was observed in the hAMs of individuals with AUD. Despite elevation of p65 in AMs due to alcohol metabolism, we showed here that EcoHIV-infection was required to trigger p65 activation in AMs. This again highlights the importance of the interaction between alcohol metabolism and HIV, confirming that none of these stressors alone was responsible for the detrimental changes in AMs. The nuclear translocation of p65 is an energy-consuming process, like HIV replication and IL-1β activation. This raises the question of whether the interplay between acetaldehyde-induced p65 activation and HIV replication is bidirectional in AMs. Additional studies may be necessary to accurately determine the molecular order of these events.

Collectively, our data provide evidence that acetaldehyde-induced p65 signaling pathway is crucial for mitochondrial hyperactivity, which in turn facilitates HIV replication and IL-1β activation. Therefore, targeting p65 activation with modulators such as PIO may be critical in restoring mitochondrial function, thereby attenuating HIV replication and inflammatory responses in AMs. We already demonstrated that PIO treatment not only replenished PPARγ depletion in HIV-infected AMs exposed to acetaldehyde but also attenuated HIV replication as well as IL-1β activation [18]. We also showed that the inhibition of p65 activity explains how PIO suppresses acetaldehyde-induced HIV replication and IL-1β activation [18]. In this study, we show that PIO restored mitochondrial bioenergetic function in acetaldehyde-exposed AMs, thereby creating an energy-deficient environment for p65-induced HIV replication and IL-1β activation. While we do not fully comprehend how PIO regulates the interplay between acetaldehyde-induced p65 and mitochondrial function, PIO has been shown to maintain mitochondrial biogenesis function by regulating molecules such as, peroxisome proliferator-activated receptor gamma coactivator 1-alpha [73,74], a negative regulator of p65 and proinflammatory activation [75]. PIO likely uses the same mechanism to attenuate p65, hence restoring mitochondrial bioenergetics in acetaldehyde-exposed HIV-infected AMs. Furthermore, it has been reported that post-translational modification by acetylation activates the p65 signaling pathway [76]. Since AGS are effective acetylating agents [42], the activation of p65 in AMs may partly result from acetylation. Additionally, PIO inhibits p65 activation by decreasing its acetylation [77]. Therefore, PIO’s regulation of p65 acetylation may also be responsible for restoring mitochondrial function as a means of inhibiting HIV replication and IL-1β activation.

PIO, which inhibits ROS production, HIV replication, and inflammatory changes, may also provide a therapeutic benefit for mitochondrial derangements in HIV-infected AMs exposed to alcohol metabolism. In fact, we demonstrated that PIO partially reversed mitochondrial dysfunction in AGS-exposed EcoHIV-infected AMs, including elevated proton leak and non-mitochondrial respiration. This is not the first study to demonstrate PIO’s potential to rescue AMs from alcohol exposure. We previously demonstrated that PIO improved mitochondrial bioenergetics after they were disrupted by alcohol in AMs [35]. More recently, we demonstrated that PIO reversed oxidative stress-induced mitochondrial dysfunction in hAMs of individuals with AUD [25]. Additionally, PIO has proven to bolster the cellular endogenous antioxidant pathway, attenuate proinflammatory responses, inhibit the p65 signaling pathway, and even restore AM immunity within the context of HIV infection and alcohol exposure [25,35,78].

## 5. Conclusions

**Figure 11:**
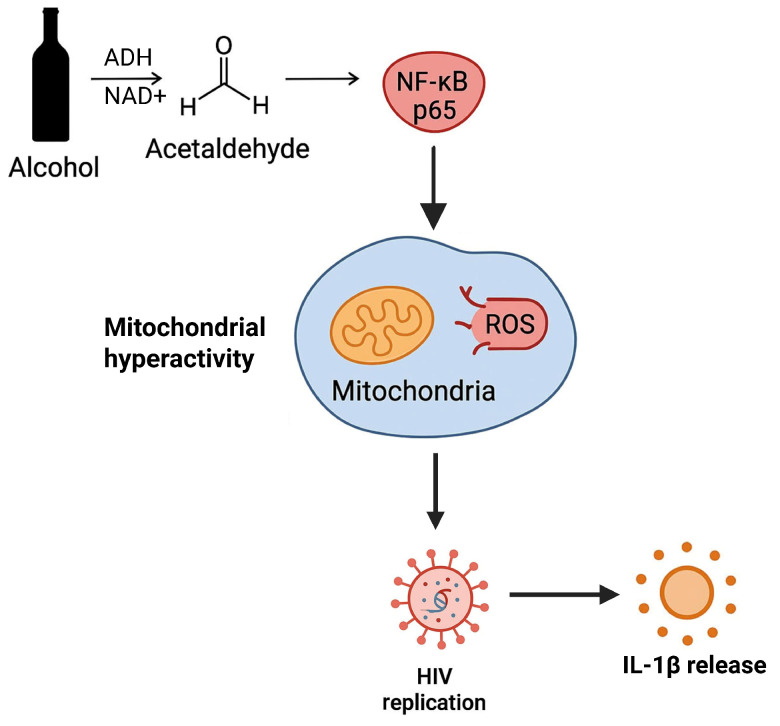
In HIV-infected AMs, acetaldehyde-induced p65 enhances ROS production and mitochondrial hyperactivity as an adaptive response to the energy demands required by HIV replication and IL-1β release.

In this study, we detected increased levels of p24 antigen in the BALF of ART-compliant PWH with undetectable viremia and elevated CD4 cell counts. We also demonstrated that p24 in the BALF of ART-compliant PWH represents replication-competent HIV in the presence of alcohol metabolism. Furthermore, we observed increased expression of p65 in hAMs of AUD individuals, and acetaldehyde enhanced p65 activation in HIV-infected primary mAMs. In HIV-infected AMs, we demonstrated that acetaldehyde-induced p65 enhanced mitochondrial hyperactivity, leading to detrimental outcomes, including ROS production, HIV replication, and IL-1β activation (Figure 11). Treating HIV-infected AMs exposed to acetaldehyde with PIO suppressed acetaldehyde-induced p65, HIV replication, and IL-1β activation by restoring mitochondrial bioenergetic functions similar to control levels.

## Figures and Tables

**Table 1:** Mouse primer sequences used for qRT-PCR to measure mRNA levels of EcoHIV constructs, *IL-1β* and *GAPDH*.

Target Genes	Forward Primer	Reverse Primer
*MLV envelope protein*	5′-TGG GAC CAC AGG CTA CAC TAGA-3′	5′-TGA TGA CAG CAT GCC AGG GAG TGG-3′
*EcoHIV gag*	5′-TGG GAC CAC AGG CTA CAC TAGA-3′	5′-CAG CCA AAA CTC TTG CTT TAT GG-3′
*EcoHIV Nef*	5′-GAG TGA AAA ATC TCT AGC AGT GGC GC-3′	5′-GCT GAA GAG GCA CAG GTT CCT CAG GTCG-3′
*EcoHIV Tat*	5′-CCT AGG ACT GCT TGT AAT AAG TGT-3′	5′-GTC GGG TCC CCT CGG GAC TGG GAG-3′
*EcoHIV Vif*	5′-AAG AGG CGA GGG GCA GCGA-3′	5′-TCT TTA CTT TTC TTC TTG GTA CTA CCT TTA TG-3′
*HIV 1 gp120*	5′-TCC TGC TCA ACT TCC TGT CGA G-3′	5′-CAC AGG TCA AAC CTC CTA GGA ATG-3′
*HIV 1 gag*	5′-GAG GAT CCC CCA TAG TGC AGA ACC TC-3′	5′ CCG GTA CCT TAG AAA ACT CTT GCT TTA TG-3′
*HIV 1 Tat*	5′-GAA GCA TCC AGG AAG TCA GC-3′	5′-GGA GGT GGG TGC TTT GAT AG-3′
*IL-1β* (human)	5′-AGC TAC GAA TCT CCG ACCAC-3′	5′-CGT TAT CCC ATG TGT CGA AGA A-3′
*GAPDH* (mouse, human, or rabbit)	5′-AGC TTG TCA TCA ACG GGA AG-3′	5′-TTT GAT GTT AGT GGG GTC TCG-3′

**Table 2:** Demographic characteristics of individuals with and without AUDs.

	Total	Non-AUD (*n* = 6)	AUD (*n* = 8)
Age in years (mean(SD))	39.78 (11.29)	39.75 (13.19)	41.33 (9.1)
Gender (%)			
Male	50%	50%	50%
Female	50%	50%	50%
Race (%)			
Black	79%	83%	75%
White	7%	17%	0
More than one race	14%	0	25%

## Data Availability

Details regarding the data supporting the reported results are available on the University of North Carolina Dataverse at https://doi.org/10.15139/S3/EKERUH (accessed on 7 November 2025).

## Supplementary Materials

The following supporting information can be downloaded at: https://www.mdpi.com/article/10.3390/biom15121737/s1, Original blots for NADPH OXIDASE (NOX) 4 and p65.

## Abbreviations

The following abbreviations are used in this manuscript:

**Abbreviations**	**Definitions**
PWH	People living with HIV
p65	Nuclear Factor Kappa B p65
IL-1β	Interleukin-1 beta
AMs	Alveolar macrophages
PIO	Pioglitazone
ROS	Reactive oxygen species
ART	Antiretroviral therapy
BALF	Bronchoalveolar lavage fluid
AIDS	Acquired immune deficiency syndrome
ATP	Adenosine triphosphate
AUD	Alcohol use disorder
PPARγ	Peroxisome proliferator-activated receptor gamma
FBS	fetal bovine serum
NOX4	nicotinamide adenine dinucleotide phosphate oxidase 4
PWoH	People living without HIV
IRB	Institutional review board
CD4	Cluster differentiation 4
MAA	Malondialdehyde-acetaldehyde
USA	United States of America
NAD^+^	Nicotinamide adenine dinucleotide
ADH	Alcohol dehydrogenase
AGS	Acetaldehyde generating system
mAMs	Mouse alveolar macrophages
PFA	Paraformaldehyde
ELISA	Enzyme linked immunosorbent assay
PMA	Phorbol 12-myristate 13-acetate
OCR	Oxygen consumption rate
FCCP	carbonilcyanide p-triflouromethoxyphenylhydrazone
MLV	Murine leukemia virus
qRTPCR	Quantitative real-time polymerase chain reaction
GAPDH	glyceraldehyde-3-phosphate dehydrogenase
mRNA	Messenger ribonucleic acids
Gp20	Glycoprotein 120
Gag	Group-specific antigen
Tat	Trans-activator of transcription
NIH	National Institute of Health
PBS	Phosphate-buffered saline
BSA	Bovine serum albumin
DAPI	4′,6-diamidino-2-phenylindole
PAMP	Pathogen-associated molecular pattern
hAM	Human alveolar macrophages
RFU	Relative fluorescence unit
ANOVA	Analysis of variance
MitoSOX	Mitochondrial superoxide
H_2_O_2_	Hydrogen peroxide
