# Supplementary material for: Pioglitazone Modulates p65-Mediated Mitochondrial Bioenergetics: Implications for Acetaldehyde-Induced HIV Replication in Alveolar Macrophages
Source: Biomolecules. 2025 Dec 13;15(12):1737. doi: 10.3390/biom15121737 (PMC12730783; doi:10.3390/biom15121737)
Supplement: Supplementary file 1 [file biomolecules-15-01737-s001.zip › biomolecules-4004265-supplementary.pdf]

ORIGINAL BLOTS FOR NADPH OXIDASE (NOX) 4 and p65

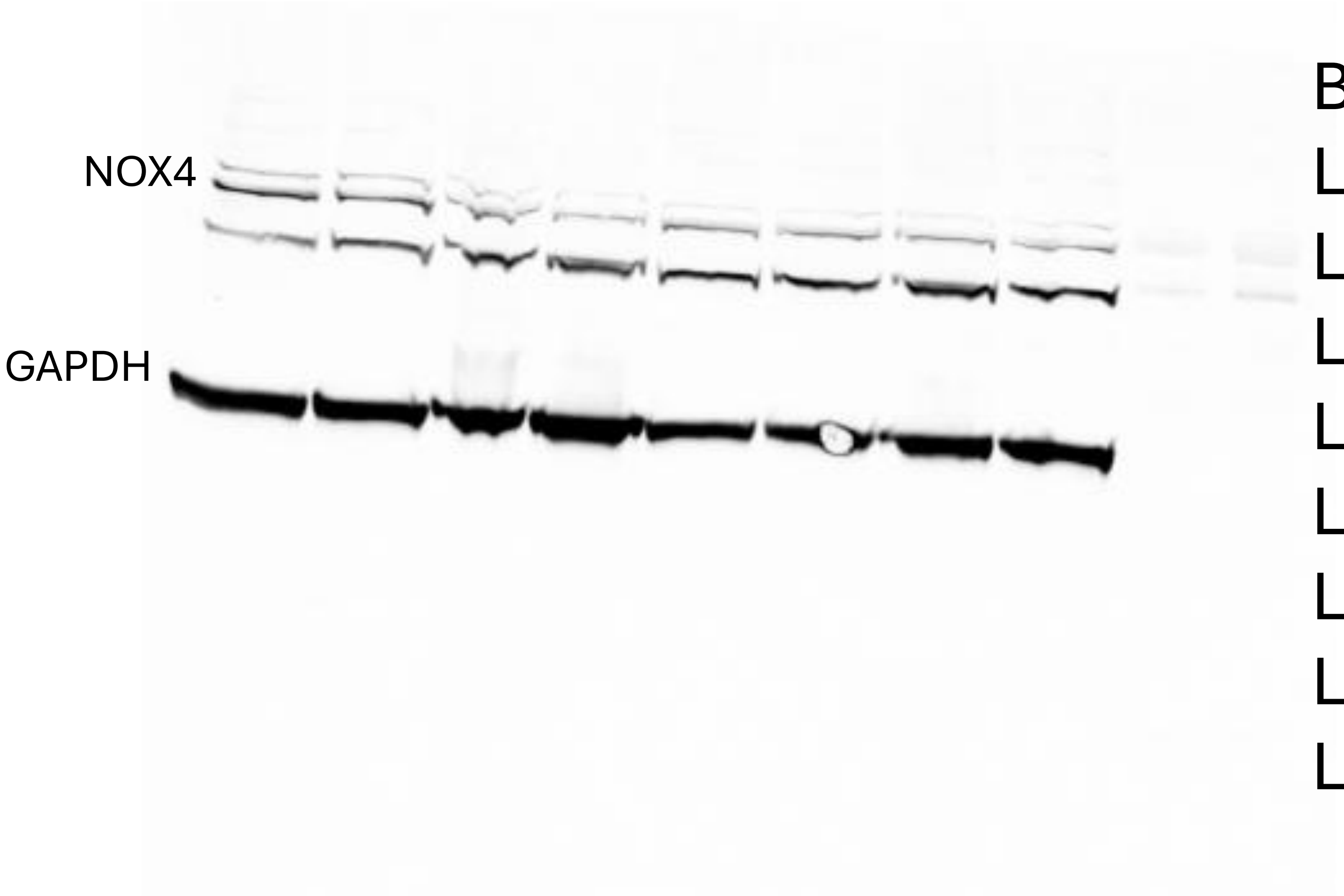

Blot is labeled from right to left:
Lane 1: Control
Lane 2: EcoHIV
Lane 3: AGS
Lane 4: AGS + EcoHIV
Lane 5: Control
Lane 6: EcoHIV
Lane 7: AGS
Lane 8: AGS + EcoHIV

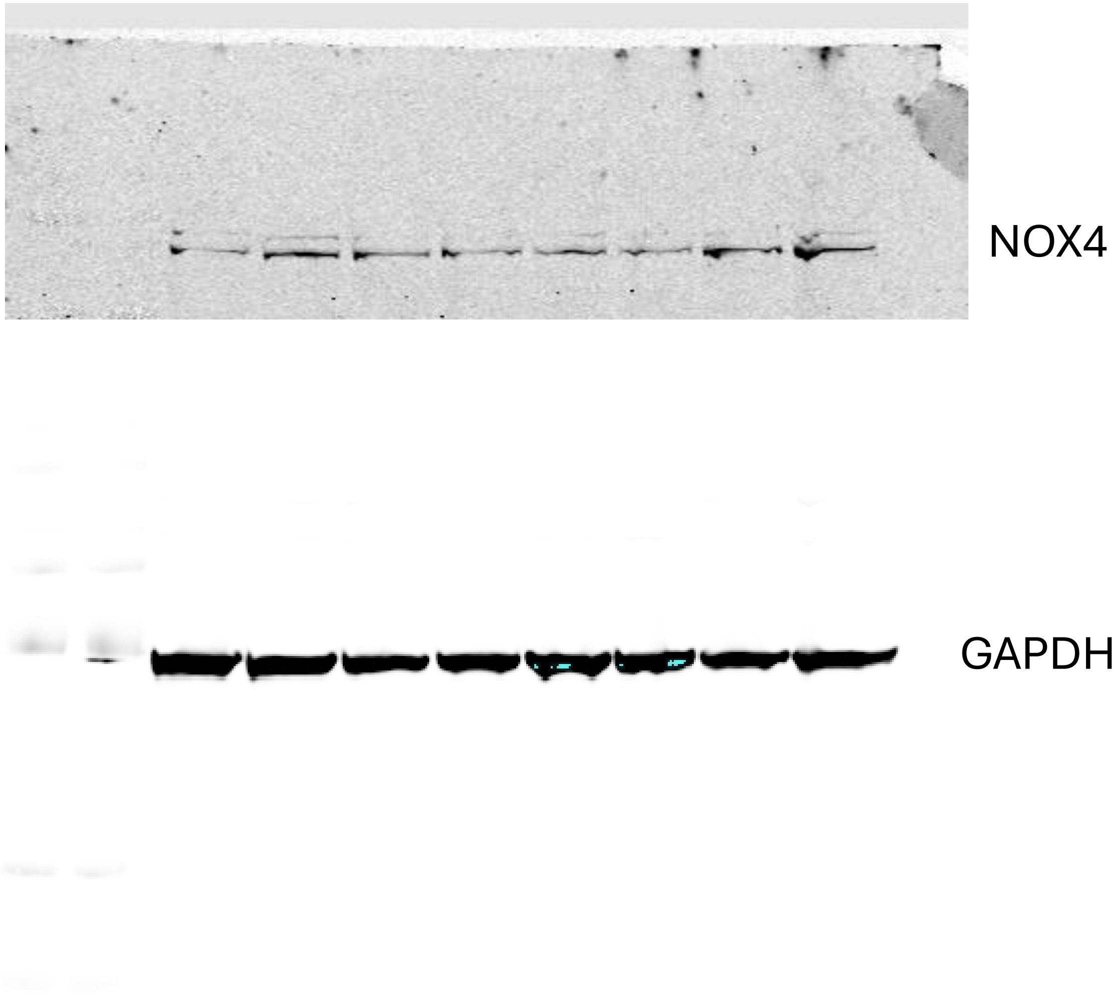

Blot is labeled from left to right:

Lane 1: Control

Lane 2: EcoHIV

Lane 3: AGS

Lane 4: AGS + EcoHIV

Lane 5: Control

Lane 6: EcoHIV

Lane 7: AGS

Lane 8: AGS + EcoHIV

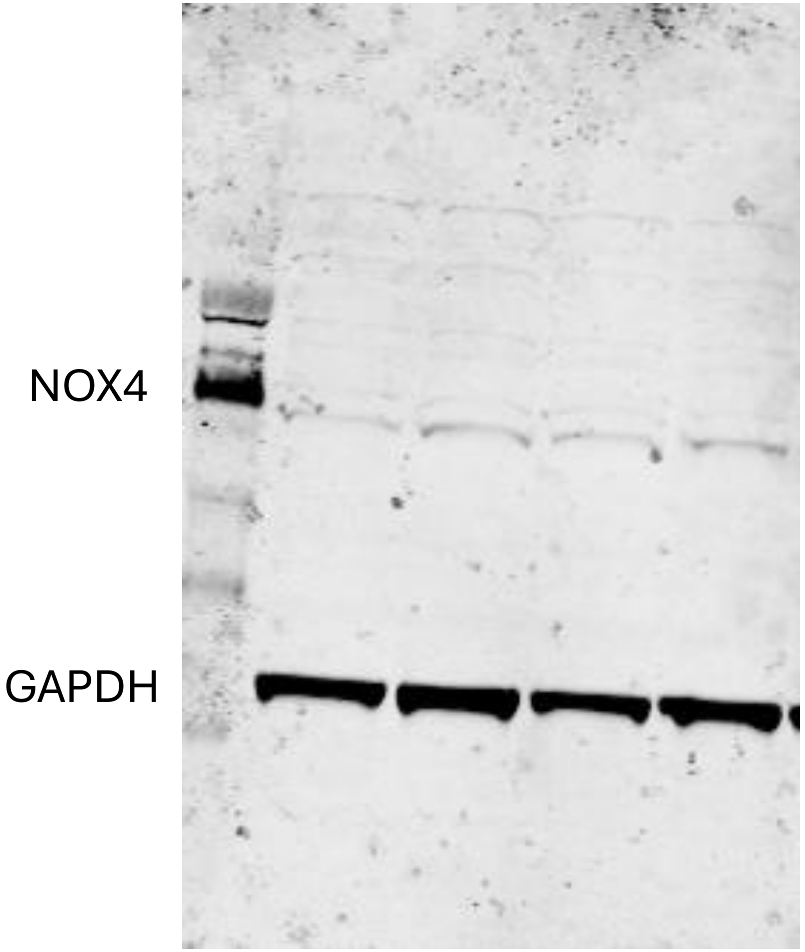

Blot is labeled from left to right:
Lane 1: Control
Lane 2: EcoHIV
Lane 3: AGS
Lane 4: AGS+EcoHIV

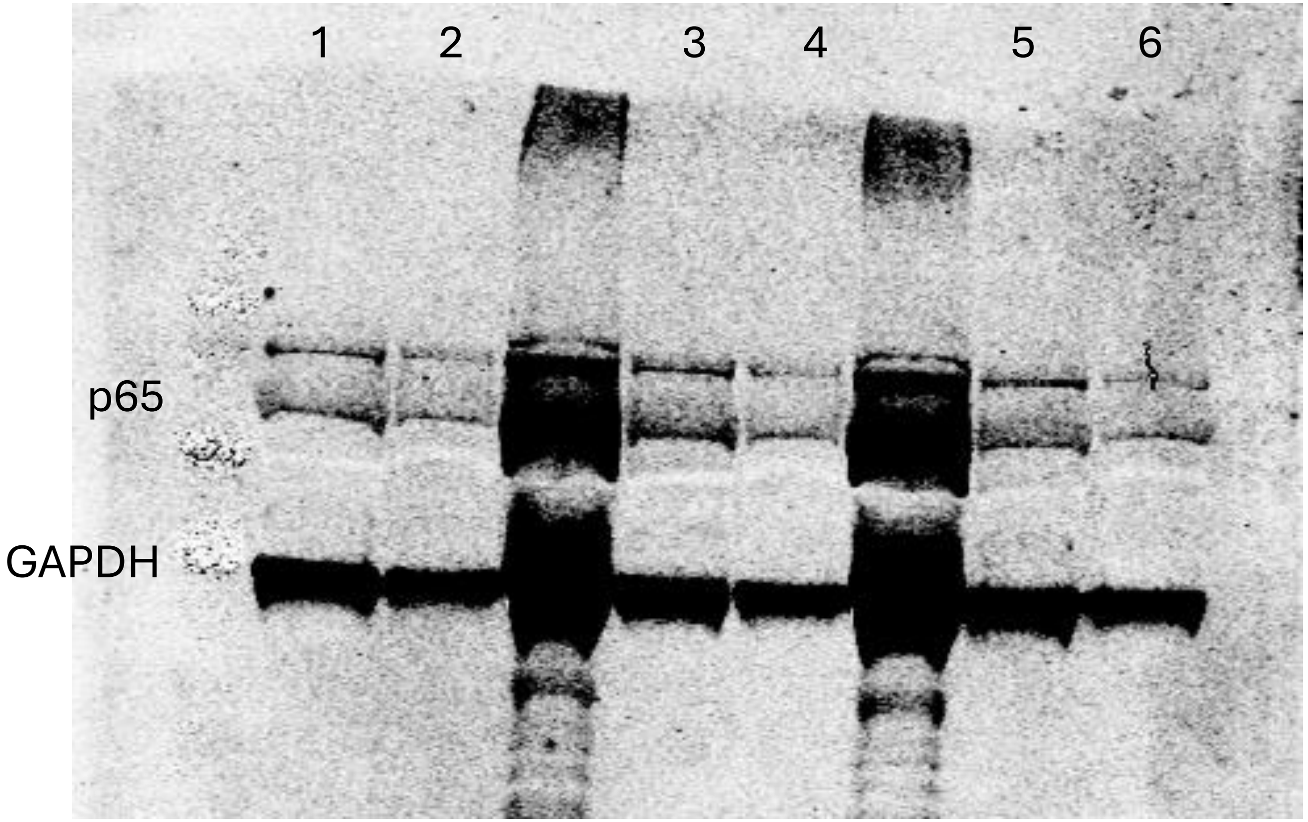

1. AGS + EcoHIV
2. AGS + EcoHIV + JSH-23
3. AGS + EcoHIV
4. AGS + EcoHIV + JSH-23
5. AGS + EcoHIV
6. AGS + EcoHIV + JSH-23

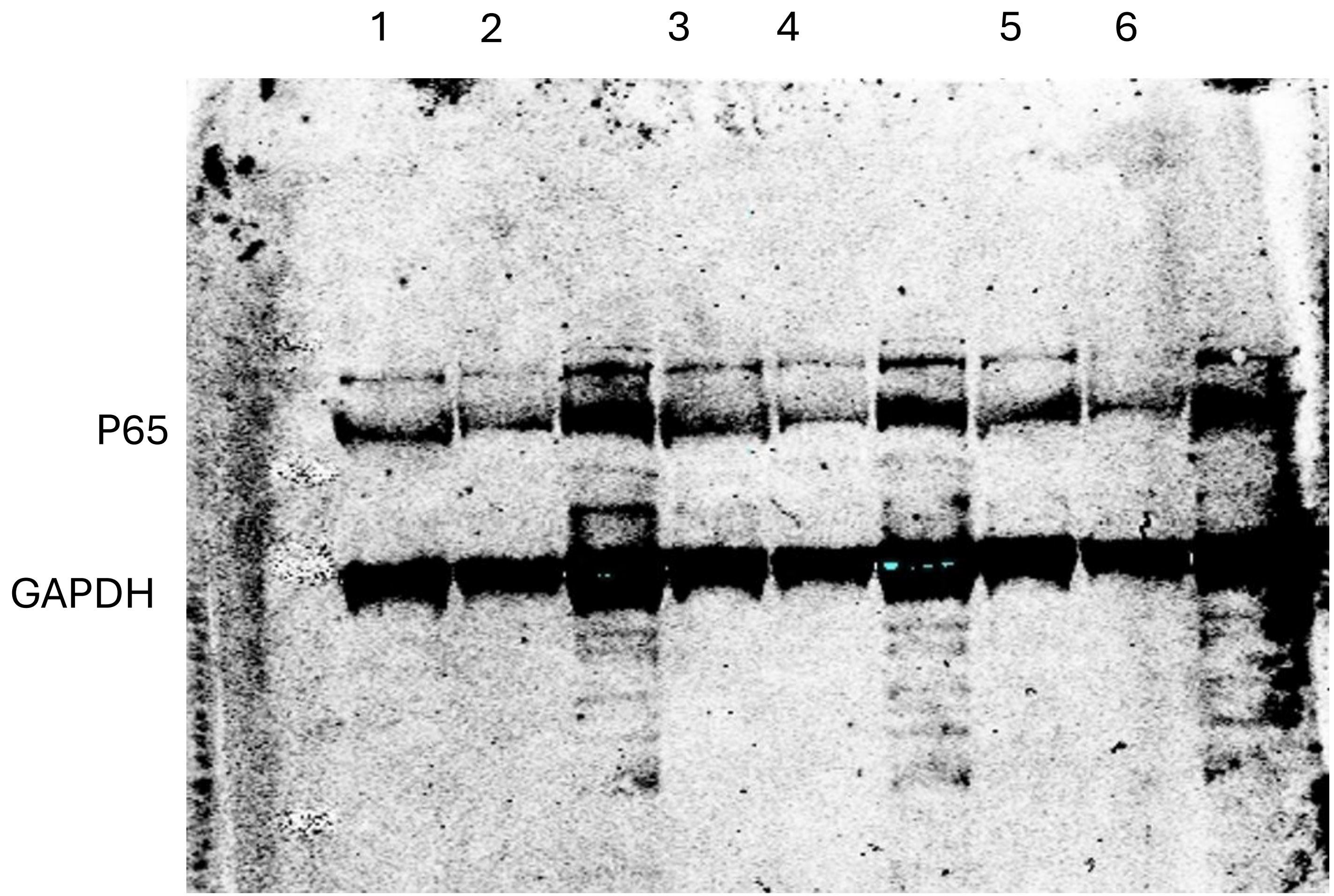

1. AGS + EcoHIV
2. AGS + EcoHIV + JSH-23
3. AGS + EcoHIV
4. AGS + EcoHIV + JSH-23
5. AGS + EcoHIV
6. AGS + EcoHIV + JSH-23
